# Orthopteran Neo‐Sex Chromosomes Reveal Dynamics of Recombination Suppression and Evolution of Supergenes

**DOI:** 10.1111/mec.17567

**Published:** 2024-10-30

**Authors:** Suvratha Jayaprasad, Valentina Peona, Simon J. Ellerstrand, Roberto Rossini, Ignas Bunikis, Olga V. Pettersson, Remi‐André Olsen, Carl‐Johan Rubin, Elisabet Einarsdottir, Franziska Bonath, Tessa M. Bradford, Steven J. B. Cooper, Bengt Hansson, Alexander Suh, Takeshi Kawakami, Holger Schielzeth, Octavio M. Palacios‐Gimenez

**Affiliations:** ^1^ Population Ecology Group Institute of Ecology and Evolution Friedrich Schiller University Jena Jena Germany; ^2^ Department of Organismal Biology–Systematic Biology Evolutionary Biology Centre Uppsala University Uppsala Sweden; ^3^ Swiss Ornithological Institute Sempach Switzerland; ^4^ Department of Biology Lund University Lund Sweden; ^5^ Department of Biosciences University of Oslo Oslo Norway; ^6^ Department of Immunology, Genetics and Pathology Uppsala Genome Center Uppsala University National Genomics Infrastructure hosted by SciLifeLab Uppsala Sweden; ^7^ Department of Biochemistry and Biophysics Science for Life Laboratory Stockholm University Solna Sweden; ^8^ Department of Medical Biochemistry and Microbiology – Disciplinary Domain of Medicine and Pharmacy Faculty of Medicine Uppsala University Uppsala Sweden; ^9^ Department of Gene Technology Science for Life Laboratory KTH‐Royal Institute of Technology Solna Sweden; ^10^ Evolutionary Biology Unit South Australian Museum Adelaide South Australia Australia; ^11^ School of Biological Sciences and Environment Institute The University of Adelaide Adelaide South Australia Australia; ^12^ School of Biological Sciences University of East Anglia Norwich Research Park Norwich UK; ^13^ Centre for Molecular Biodiversity Research Leibniz Institute for the Analysis of Biodiversity Change, Zoologisches Forschungsmuseum A. Koenig Bonn Germany; ^14^ Embark Veterinary, Inc. Boston Massachusetts USA; ^15^ German Centre for Integrative Biodiversity Research (iDiv) Halle‐Jena‐Leipzig Leipzig Germany

**Keywords:** chromosomal rearrangements, genetic degeneration, genomic recombination, neo‐sex chromosomes, repetitive DNA, sexual antagonistic locus, supergenes

## Abstract

The early evolution of sex chromosomes has remained obscure for more than a century. The *Vandiemenella viatica* species group of morabine grasshoppers is highly suited for studying the early stages of sex chromosome divergence and degeneration of the Y chromosome. This stems from the fact that neo‐XY sex chromosomes have independently evolved multiple times by X‐autosome fusions with different autosomes. Here, we generated new chromosome‐level assemblies for two chromosomal races representing karyotypes with and without neo‐sex chromosomes (P24XY and P24X0), and sequence data of a third chromosomal race with a different neo‐XY chromosome system (P25XY). Interestingly, these two neo‐XY chromosomal races are formed by different X‐autosome fusions (involving chr1 and chrB, respectively), and we found that both neo‐Y chromosomes have partly ceased to recombine with their neo‐X counterpart. We show that the neo‐XY chromosomes have diverged through accumulation of SNPs and structural mutations, and that many neo‐Y‐linked genes have degenerated since recombination ceased. However, the non‐recombining regions of neo‐Y chromosomes host non‐degenerated genes crucial for sex determination, such as *sex‐lethal* and *transformer*, alongside genes associated with spermatogenesis, fertility, and reproduction, illustrating their integrative role as a masculinizing supergene. Contrary to expectations, the neo‐Y chromosomes showed (slightly) lower density of transposable elements (TEs) compared to other genomic regions. The study reveals the unique dynamics of young sex chromosomes, with evolution of recombination suppression and pronounced decay of (some) neo‐sex chromosome genes, and provides a compelling case illustrating how chromosomal fusions and post‐fusion mutational processes contribute to the evolution of supergenes.

## 1 Introduction

Sex chromosomes typically evolve from an ancestral pair of autosomes that acquire a master sex‐determining locus and subsequently lose their ability to recombine (Charlesworth 2017; Ohno 1967; Wright et al. 2016). The lack of recombination has critical consequences for sex chromosome evolution, impairing the efficacy of natural selection and facilitating substantial divergence between the X‐Y (or Z‐W) pairs by chromosome rearrangements. Furthermore, the lack of recombination leads to an accumulation of repetitive sequences, loss of genetic diversity, and genetic degeneration on the Y (or W) (Charlesworth 2017; Ohno 1967; Wright et al. 2016). Empirical evidence on sex chromosome degeneration mostly comes from species with highly diverged (ancient) sex chromosomes, as found in mammals (Lahn and Page 1999; Skaletsky et al. 2003), birds (Bellott et al. 2017; Yazdi, Silva, and Suh 2020), and *Drosophila* (Bachtrog 2013). Data from these study systems show that their sex chromosomes evolved from ordinary autosomes through extensive evolutionary processes, including stepwise recombination suppression forming evolutionary strata, where recombination has ceased at different time points (Lahn and Page 1999; Skaletsky et al. 2003). Although empirical approaches to study genetic degeneration have been developed for model organisms (Bachtrog 2013; Bellott et al. 2017; Lahn and Page 1999; Skaletsky et al. 2003), the onset and dynamics of these changes are still poorly understood primarily due to the lack of study systems with populations in a transition from autosomes to sex chromosomes (Charlesworth, Charlesworth, and Marais 2005; Charlesworth and Charlesworth 1980). However, sex chromosomes sometimes fuse with autosomes to form so‐called neo‐sex chromosomes, providing a fresh start to sex chromosome evolution.

Since neo‐sex chromosomes are comparatively young, we expect to find traces of their pre‐fusion identities, a pattern that is often referred to as the genetic footprints (Ming et al. 2021). Fusion involving a sex chromosome and an autosome creates an imbalance with unequal partners. Typically, the neo‐X retains more genes crucial for both sexes, while the neo‐Y undergoes mutation accumulation and gene loss (Huang et al. 2022; Nozawa et al. 2021). The formation of neo‐sex chromosomes can sometimes shuffle genes associated with reproduction and mate selection, thereby influencing speciation processes by creating barriers to interbreeding among closely related populations (Wang et al. 2022). Investigating the evolutionary dynamics of neo‐sex chromosomes can thus provide unique insights into the early evolutionary processes during divergence of sex chromosomes from their autosomal origin (Charlesworth, Charlesworth, and Marais 2005; Sigeman et al. 2021) and allow the aspects of the early evolution of sex chromosomes to be better understood.

The majority of Orthoptera species (grasshoppers, crickets, and bush‐crickets) has an X0 sex‐determining system, with X0 in males and XX in females, which is believed to have originated from the loss of the Y chromosome in an ancestral XY/XX species (Castillo, Marti, and Bidau 2010). Degeneration of the Y has led to the translocation of functional genes to autosomes and ultimately to the complete loss of the Y chromosome. However, despite X0 being ancestral in Orthoptera, Y chromosomes still exist in several groups suggesting that Y chromosomes have arisen *de novo* (Castillo, Marti, and Bidau 2010). In grasshoppers of the subfamilies Morabinae and Melanoplinae, for example, derived variants like neo‐XY in males and neo‐XX in females evolved several times by repeated X‐autosome (X‐A) chromosome fusions (Castillo, Marti, and Bidau 2010; White 1978). Sexually antagonistic selection may promote the fixation of X‐A fusions if the fused autosome harbors sex‐specific alleles that thereby become tightly linked to the sex‐determining locus (Pannell and Pujol 2009; Ponnikas et al. 2018; Veltsos, Keller, and Nichols 2008). Suppression of recombination between the ancestrally identical autosomes may then permanently link coadapted alleles into supergenes, inherited as a single genetic element, facilitating complex phenotypic adaptation (Hendrickx et al. 2021; Joron et al. 2011; Küpper et al. 2016). Mounting evidence suggests that supergenes play a pivotal role in the evolution of sex, ecotypes, cryptic morphs, and social organization (Hendrickx et al. 2021; Joron et al. 2011; Küpper et al. 2016).

Neo‐sex chromosomes may resolve intralocus sexual conflicts by allowing sexually antagonistic alleles to segregate in association with the sex‐determining locus (Bull 1983; Fisher 1931; Rice 1984). This can strongly favor the spread of the neo‐sex chromosomes (Ponnikas et al. 2018; Veltsos, Keller, and Nichols 2008). Because of the male‐limited inheritance of neo‐Y chromosomes and the typically stronger sexual selection in males, selection for suppression of recombination is likely to be stronger for neo‐Y than for neo‐X chromosomes. The non‐recombining part of the neo‐Y chromosome would then become vulnerable to gene decay, accumulation of deleterious mutations and repetitive DNA, and ultimately to extensive chromosome divergence (Charlesworth 2017, 2021a). However, the patterns of recombination suppression and gene decay are not universal, and it remains unclear how and why recombination is halted (Filatov 2022; Ponnikas et al. 2018; Sigeman et al. 2021; Yazdi, Silva, and Suh 2020), particularly in newly evolved neo‐sex chromosomes like those found in Orthoptera.

The karyotypically diverse Australian morabine grasshoppers (Morabinae) of the genus *Vandiemenella* form a species complex with an estimated divergence time < 0.5–3.1 Myr based on mitochondrial markers (Kawakami et al. 2009). The *Vandiemenella viatica* species group contains two nominal species (*Vandiemenella pichirichi* and *Vandiemenella viatica*) and seven provisional races/species (*viatica*19, *viatica*17, P24, P25, P45b, P45c, and P50) differentiated by extensive chromosome rearrangements, including centric fusions, fissions, translocations, and inversions (Kawakami et al. 2009; White 1978). Remarkably, centric fusions of the ancestral X chromosome occurred with different autosomes in at least three instances (P24X0/XY, P25X0/XY, and P45bX0/XY races) (Kawakami et al. 2009; White 1978) resulting in three evolutionarily independent cases of newly evolved neo‐XY sex chromosomes. The dynamic nature of the sex chromosomes in the *viatica* species group makes the group highly suited for studying the processes involved in the evolution of male‐limited chromosomes. Different genes became Y‐linked in different species, providing a unique opportunity to assess the effects of male limitation. This can provide new insights into why neo‐sex chromosomes evolved in the first place, how fast neo‐sex chromosomes diverge, and why gene degeneration continues.

**FIGURE 1:**
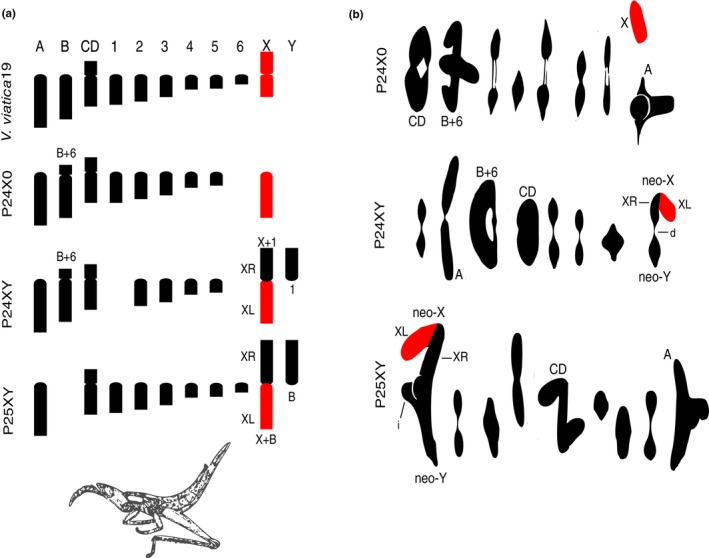
Male haploid karyotypes, hypothesized chromosomal changes, and meiosis observed within the analyzed chromosomal races of the *Vandiemenella* morabine grasshoppers. (a) Overview of male haploid karyotypes and hypothesized chromosomal changes of the morabine grasshoppers *V. viatica*19, P24X0, P24XY, P25XY, based on White (1978). Chromosomes are sorted by size and centromere position. Noteworthy are two instances of independent neo‐XY sex chromosome formation via centric fusion events between the ancestral X chromosome (red) and one of the autosomes (black). (b) First metaphases of males in P24X0, P24XY and P25XY, modified from White, Blackithr, and Cheney (1967). The largest autosome pairs, the sex chromosomes, and chromosome arms of the neo‐sex chromosomes involved in X‐A centric fusions are indicated. XL refers to the arm originating from the ancestral X chromosome fused to an autosome, while XR designates the autosomal arm of the neo‐X that shares homology with the neo‐Y. The location of chiasmata between neo‐XR and neo‐Y in P24XY (d: Distal) and P25XY (i: Interstitial) is specified.

Based on previous cytological analysis (White 1978; White, Blackithr, and Cheney 1967), the karyotype of *viatica*19 (*n* = 9 + X0) is regarded as ancestral for the group (Figure 1a). It comprises two pairs of acrocentric autosomes (designated as chrA and chrB), an unequal‐armed metacentric autosome pair chrCD, six pairs of small acrocentric autosomes (designated as chr1 to chr6), and the metacentric X chromosome. Autosome chr6 is characterized by being late‐replicating and entirely heterochromatic in all members of the group (Webb and White 1975). The P24 race (*n* = 8 + X0) differs karyotypically from *viatica*19 in possessing a centric fusion of the large autosome chrB with small acrocentric autosome chr6. P24X0 also differs from *viatica*19 in that the X chromosome is acrocentric instead of metacentric, likely owing to a pericentric inversion involving the centromere (White 1978). P24XY (*n* = 7 + neo‐XY) differs from P24X0 in that the acrocentric X has undergone a centric fusion with the autosome chr1, resulting in the formation of a neo‐XY system (White 1978). The unfused chr1, now exclusive to males, becomes a neo‐Y chromosome, with the neo‐X chromosome designated as XL for the arm derived from the original X chromosome, and as XR for the arm sharing homology with the neo‐Y (White 1973). The race P25XY (n = 8 + neo‐XY) has a centric fusion between the X and the chrB, thereby also giving rise to a different neo‐XY system (White 1978).

It is well stablished that orthopteran chromosomes undergo recombination during both male and female meiosis (Hewitt 1979; Perry and Jones 1974; Viera et al. 2004; White 1973). The meiotic behavior of the X chromosome in the morabine grasshopper X0 races is readily identifiable (Figure 1b). It exists as a single, unpaired, and achiasmatic (non‐recombining) element (White, Blackithr, and Cheney 1967). During male meiosis in P24XY (Figure 1b), the neo‐Y consistently pairs with the XR arm, typically forming a single chiasma between them, which usually terminates before the first metaphase (White, Blackithr, and Cheney 1967). The P25XY neo‐Y appears to be fully homologous to the XR during meiosis (Figure 1b); the chiasma formed between them often does not completely terminalize, remaining interstitial during the first metaphase (White, Blackithr, and Cheney 1967). These cytogenetic patterns suggest that a segment of the neo‐Y in P24XY and P25XY does not undergo recombination, although the exact extent and degree of recombination suppression remain uncertain.

Here, we report on the analysis of chromosome‐level annotated genomes of two chromosomal races representing two pairs of karyotypes with and without neo‐sex chromosomes (P24 XY and X0) along with sequence for the race P25XY. We investigate the chromosomal structure of the neo‐sex chromosomes and how previously autosomal regions have evolved in the new sex‐linked environment in terms of recombination suppression, repeat accumulation, and gene differentiation. Finally, we discuss how this neo‐sex chromosome diversification contributed to the diversification of the *Vandiemenella viatica* species complex.

## 2 Materials and Methods

### 2.1 Sampling, DNA Extraction, and Sequencing

Chromosomal races of morabine grasshoppers in the *Vandiemenella viatica* species group (P24X0, P24XY, and P25XY) were collected between 2002 and 2017 in southern Australia. Races/species were identified by either karyotyping (males) or based on 10 morphometric characters of the female reproductive structures that were unambiguously distinguishable from the other chromosomal races (Atchley and Cheney 1974). Testes were dissected and fixed for karyotyping as described previously (Kawakami et al. 2009; White et al. 1982). The remaining body parts were flash frozen in liquid nitrogen and stored at −80°C in the Australian Biological Tissue Collection until DNA extraction. DNA extraction was done from either heads or legs using the MagAttract HMW DNA Kit (QIAGEN, Hilden, Germany; Cat No. 67563).

A total of 9 males and 13 females of the morabine grasshoppers were sequenced using different platforms: (i) We sequenced the genome of one female P24XY using PacBio long‐read sequencing (54 Sequel SMRT cells 1 M on a PacBio Sequel system), which produced 315 Gb of raw data (genome coverage 84x) with insert N50 size of 8.2 Kb; (ii) two females of P24XY were used to produce two paired‐end libraries (150 bp read length) of chromatin conformation capture (Hi‐C) data from Arima Genomics, sequenced using Illumina HiSeq 2500 that produced 440 million reads; (iii) we sequenced one female of P24XY using 10X Genomics Chromium linked‐read with the illumina HiSeqX machine 22.3 kb mean molecule length, 380 bp library insert size, 150 bp read length, 1784.29 million reads, net coverage 78.3× estimated by Supernova 2.1.0; (iv) we re‐sequenced 3 males and 3 females each of P24X0, P24XY, and P25XY on a Illumina NovaSeq 6000 machine (2× 150 bp read length) to ~20x genome coverage; (v) we used existing 10X Genomics Chromium linked‐read libraries (males P24X0/P24XY), and RNA‐seq reads from males and females (heads, legs, testis and ovaries) of P24X0/P24XY races, previously generated elsewhere (Palacios‐Gimenez et al. 2020) (BioProject PRJNA668746). The data generated and used are listed in Table S1.

### 2.2 Genome Assembly

#### 2.2.1 Female P24XY Reference Assembly

We assembled the PacBio long‐read sequencing into a phased primary assembly using Falcon unzip (Chin et al. 2016) followed by polishing with Arrow (Chin et al. 2013; Koren et al. 2017). We masked repeat regions of the phased primary assembly using a custom repeat library (Palacios‐Gimenez et al. 2020) and then polished with two rounds in Pilon 1.24 (Walker et al. 2014) with the parameter “‐‐fix indels” using the generated P24XY female 10X Genomics Chromium linked‐read. The haplotigs were removed with purge_dups v1.2.6 (Guan et al. 2020). We used the 3D‐DNA pipeline (Dudchenko et al. 2017) to join contigs into chromosomes, the juicer 1.6 pipeline (Durand et al. 2016) to map the generated P24XY female Hi‐C data, and the asm pipeline (Dudchenko et al. 2017) to do the scaffolding. Finally, we corrected misassemblies through the visual inspection of the contact map with juicebox v1.11.08 (Durand et al. 2016). The final chromosome‐level assembly was polished with two rounds in Pilon as described above. We refer to this chromosome‐level assembly as the “reference genome.”

#### 2.2.2 P24X0 Assembly

We used our existing male P24X0 Supernova draft linked‐read assembly (Palacios‐Gimenez et al. 2020) to produce reference‐guided pseudochromosomes employing RaGOO v1.11 (Alonge et al. 2019) together with the female P24XY reference genome. Before scaffolding, we removed duplicate contigs of at least 99% identity in the assemblies using dedupe procedure in BBTools (https://sourceforge.net/projects/bbmap/). We assessed the completeness of the newly generated chromosome‐level assemblies using BUSCO with the insecta_odb10 data (*n* = 1367) in BUSCO v.5.2.2 (Manni et al. 2021).

### 2.3 Genome Size Estimation

Genome size for the race P25XY was directly estimated from sequencing reads using a k‐mer‐based approach in GenomeScope 2.0 (Ranallo‐Benavidez, Jaron, and Schatz 2020). The k‐mers in fastq.gz files were counted with squeakr v0.7 (Pandey et al. 2018) with the setting “‐e ‐k 21 ‐s 36 ‐t 16.”. A list of k‐mers was obtained with the command “squeakr list,” converted into a histogram using a custom script (https://github.com/octpalacios/kmer_list_to_hist), and then parsed to GenomeScope for the genome size estimation.

### 2.4 Genome Annotation

We used species‐specific repeat prediction to best annotate repeats in the newly generated chromosome‐level assemblies. RepeatModeler2 (Flynn et al. 2020) was used to *de novo* predict and classify repeats and compile assembly‐specific repeat libraries. We then merged each generated repeat library with existing ones for *viatica* grasshoppers (Palacios‐Gimenez et al. 2020) using ReannTE_MergeFasta.pl. https://github.com/4ureliek/ReannTE with the parameter “‐s 80,” and given priority setting to favor the repeat libraries generated from the chromosome‐level assemblies when choosing consensus sequences to keep. We then concatenated the newly generated libraries (P24X0 and P24XY) with the Arthropoda consensus sequences from Repbase (Bao, Kojima, and Kohany 2015), plus the detected satDNAs (see below), and used this as a final custom library to mask all of the newly generated chromosome‐level assemblies with RepeatMasker 4.1.0 (Smit and Hubley 2019). The final custom library and notes on their classification are given in the Data S1.

We further applied dnaPipeTE (Goubert 2023; Goubert et al. 2015) and RepeatExplorer2 (Novák, Neumann, and Macas 2020), which perform *de novo* assembly of repeats directly from short sequence reads followed by classification/quantification of TEs and satDNAs for comparative analyses. RepeatExplorer2 was run separately on male short sequence reads of each race with default parameters. dnaPipeTE was run separately on males and females using the custom repeat library together with the detected satDNA as option for annotation and quantification (settings “‐genome_size ‐genome_coverage 0.1 ‐sample_number 2 ‐RM_lib”). For comparative analyses of the races with neo‐sex chromosomes (P24XY and P25XY), we further focused on differences between sexes to obtain sequences enriched on neo‐sex chromosomes. Sequences enriched on the neo‐Y are expected to be more represented in male (neo‐XY) reads than in female (neo‐XX) reads, while sequences enriched on the neo‐X would be more represented in female than in male reads. We estimated differential enrichment of repeats between sexes by normalizing the abundance of a given repeat in the male genome by the female genome and vice versa. We used 1.1 times more abundant as the threshold for repeat enrichment as described elsewhere (Ferretti et al. 2020).

The repeat‐masked genomes of the newly generated chromosome‐level assemblies were used for the gene model annotation with the GeMoMa pipeline (Keilwagen et al. 2018). GeMoMa uses the annotation of protein‐coding genes in a reference genome to infer the annotation of protein‐coding genes in a target genome. It thereby retrieves amino acid sequence and intron position conservation. GeMoMa further allows to incorporate RNA‐seq evidence for splice site prediction. Existing paired‐end RNA‐seq reads from several individuals (head, gonad, leg) of the P24X0 and P24XY chromosomal races (Palacios‐Gimenez et al. 2020) (BioProject PRJNA668746) were first aligned to their respective newly generated chromosome‐level assemblies using HiSat2 2.2.1 (Kim, Langmead, and Salzberg 2015) with the “‐‐dta” parameter on default setting (alignment rate 92.07% and 88.60% respectively). The generated BAM alignment files were then sorted with SAMtools 1.14 (Danecek et al. 2021) and used to run GeMoMa together with *Drosophila melanogaster* Release 6 (GCA_000001215.4), *Caenorhabditis elegans* (GCA_000002985.3), *Gryllus bimaculatus* (GCA_017312745.1), *Locusta migratoria* (LocustBase, https://159.226.67.243/download.htm), *Tribolium castaneum* (GCF_000002335.3.), and *Daphnia pulex* (GCA_900092285.2) genome and annotation files as references for the homology‐based gene prediction in each of the assemblies. We used InterProScan 5.52–86.0 (Jones et al. 2014) for genome‐scale protein‐coding genes function classification.

### 2.5 Sex Chromosome Analysis

#### 2.5.1 Identifying Sex‐Linked Scaffolds

We employed the findZX pipeline (Sigeman, Sinclair, and Hansson 2022) to identify and visualize sex‐linked regions by analyzing variation in genome coverage and heterozygosity between males and females. The analysis used short reads of four individuals per sex of P24X0 and P24XY, and three individual per sex of P25XY. Sequences of P24X0 and P24XY individuals were mapped separately onto their respective assemblies, while sequences of P25XY individuals were mapped onto the reference genome of P24XY. Using the basic script (findZX), the newly generated genomic DNA Illumina short reads were trimmed, subsampled to 10x coverage, aligned to a genome assembly, and duplicated reads were removed. After removing duplicates from the BAM files, we filtered out reads based on their similarity to the reference genome. The remaining high‐quality reads were stored in new BAM files. The mismatch settings were no filtering (unfiltered), intermediate filtering (≤ 2 mismatches allowed), and strict filtering (0 mismatches allowed). Analyzing various mismatch settings can unveil valuable insights into the degree of sex chromosome differentiation and the extent of Y chromosome degeneration. Subsequently, genome coverage was calculated for each sample from the BAM files across different genome window sizes (i.e., 100 kb, 500 kb and 1 Mb). Variants were called from the “unfiltered” BAM files, followed by quality filtering and heterozygosity calculations across the different genome window sizes for each sample. Lastly, mean statistics for sex‐specific genome coverage and heterozygosity were computed (since more than one sample per sex was used), both for each chromosome/scaffold in the genome assembly and across different genome window sizes. Analyzing sex differences in genome coverage and heterozygosity is a robust and commonly used method for identifying sex chromosome systems with different levels of differentiation (Palmer et al. 2019; Sigeman, Sinclair, and Hansson 2022).

#### 2.5.2 Gametolog Extraction and Recombination Suppression Analysis

We used the PhaseWY pipeline (https://github.com/sjellerstrand/PhaseWY.git, uploaded upon publication) for phasing the P24XY and P25XY genomic data overall, with a specific focus on homologous sex chromosome sequences. PhaseWY operates independently per scaffold and progresses through several key steps. First, it identifies callable regions of the genome, followed by classification of sex‐linked regions based on differences in sex‐specific genome coverage. Subsequently, it subsets variants per scaffold, conducts read‐based phasing with WhatsHap v1.7 (Martin et al. 2016), and performs statistical phasing with SHAPEIT v4.1.3 (Delaneau et al. 2019). It then identifies sex‐linked regions based on clustering of phased haplotypes. Finally, it regenotypes the heterogametic sex to haploid genotypes separately for the X‐ and Y‐linked regions, respectively. The pipeline summarizes genomic regions in bed format as autosomal, homogametic, and heterogametic, while also outputting phased variants in vcf format for the corresponding regions.

We used four individuals per sex of P24XY and three individual per sex of P25XY for phasing. To run PhaseWY, we used the unfiltered BAM files generated with findZX and called the genotypes using freebayes v1.3.2 (Garrison and Marth 2012) with the following flags: ‐‐min‐repeat‐entropy 1 ‐‐no‐partial‐observations ‐‐use‐mapping‐quality ‐m 3 ‐q 13 ‐‐genotype‐qualities ‐‐strict‐vcf ‐‐report‐monomorphic. We employed the vcfallelicprimitives tool from the vcflib v1.0.1 toolbox (Garrison and Marth 2012) to break down complex variants into standard SNP and indel representations. The resulting variants were then normalized with Vt v0.5772‐60f436c3 (Tan, Abecasis, and Kang 2015). Subsequently, a series of filtering steps were implemented using vcflib and BCFtools version 1.9 (Danecek et al. 2021) to discard variants of low quality and those likely arising from copy number variations or paralogous sequences absent in the reference genomes. This filtering process entailed the exclusion of variants of low quality, read depths < 3 reads, and quality scores < 30. Additionally, heterozygous variants with depths < 4 and homozygous variants with quality scores < 50 and depths < 4 were excluded. For heterozygous variants, those with quality scores < 2 fold the average depth and where the maximum genotype depth exceeded the average depth plus 3 folds the square root of the average depth were also removed (H. Li 2014). Furthermore, variants occurring within repeat regions were eliminated from the file by using the RepeatMasker output file. The final filtered vcf files were used as input for PhaseWY.

We use White's (1973) terminology to refer to the arms of the neo‐X chromosome as XL for the arm derived from the original X chromosome, and as XR for the arm that shares homology with the neo‐Y. Protein‐coding genes were retrieved from the GFF annotation files after gene prediction with GeMoMa. We extracted neo‐XR and neo‐Y sequences for each protein‐coding gene of all gametologous gene pairs from males and females within the P24XY and P25XY chromosomal races. This was done using the phased and classified PhaseWY corresponding to the heterogametic genomic regions. Neo‐XR and neo‐Y gene pairs were aligned with MUSCLE v5.1 (Edgar 2004) with default options. Neo‐XR and neo‐Y gene pairs were assigned to the sex‐linked regions and the pseudo‐autosomal regions (PARs) by their coordinates in the annotation files.

Pairwise substitution rates (dN/dS) between neo‐XR and neo‐Y gene pairs were calculated with codeml from the PAML v4.10.6 package (Yang 2007). dS is considered nearly neutral since it does not alter the encoded proteins (Charlesworth 2021a; Lahn and Page 1999; Skaletsky et al. 2003). Thus, dS values can serve as a proxy for the evolutionary time elapsed since gene pairs started differentiating into neo‐XR and neo‐Y. We retained genes with dS <  2 and dN/dS < 10, as values of dN or dS > 2 may suggest saturation of substitutions and result in inaccurate estimates (Villanueva‐Cañas, Laurie, and Albà 2013). We further inferred the levels of gene degeneration within sex‐linked neo‐Y regions by tallying the number of pseudogenes likely stemming from gene duplication or retrotransposition events. The definition of a pseudogene can be somewhat ambiguous since confirming nonfunctionality is more challenging than confirming functionality (Z. Zhang et al. 2006). We therefore classified neo‐XR and neo‐Y pseudogene sequences as functional (with intact ORF) or nonfunctional (without intact ORF) based on the presence/absence of stop codons and frameshifts. For this, we used PseudoPipe (Z. Zhang et al. 2006) to perform tblastn and subsequently parsed the output to the Pseudogene v2.0.0 pipeline (Lloyd et al. 2018). The proportion of nonfunctional genes was used as a proxy of the levels of gene degeneration in each sex‐linked region.

### 2.6 Chromosomal Rearrangement

For visualizing the pairwise whole alignment of two sequences between assemblies, such as the autosomes and neo‐sex chromosomes, we used unimap 0.1(r41), a fork of minimap2 optimized for assembly‐to‐reference comparison (H. Li 2018). The script *sam2delta.py* from RaGOO (Alonge et al. 2019) was used for SAM to nucmer‐delta format conversion. Small and lower quality alignments were filtered using delta‐filter tool from the MUMmer v4.0.0rc1 toolbox (Marçais et al. 2018) with the parameters “‐m ‐i 95 ‐l 2000,”, and the filtered delta files were converted to TSV format using the show‐coords tool from the MUMmer package. We used SyRI v1.6.3 (Goel et al. 2019) with default parameters to identify genomic rearrangements in sex chromosomes and their ancestral counterparts. This analysis was conducted on pairwise whole alignments produced using unimap and MUMmer. All statistical analyses were ran in R version 4.2.2 (R Core Team 2022).

## 3 Results

### 3.1 Chromosome‐Level Genome Assembly

We assembled a reference genome using PacBio long reads and high‐throughput chromatin conformation capture (Hi‐C) long‐range scaffolding of a female from the P24XY race. More than 90% of the contig sequences of the reference genome were scaffolded into 9 chromosome models consistent with the known haploid chromosome number (White 1978). Furthermore, we used our existing draft‐linked‐read male P24X0 assembly (Palacios‐Gimenez et al. 2020) to generate and additional reference‐guided chromosome‐level assembly using the newly generated reference genome. The chromosome‐level assembly of P24X0 (*n* = 8 + X0) was also consistent with the haploid karyotype description (White 1978). Chromosome‐level assemblies spanned 3.75–3.93 Gb resulting in scaffold N50 of 389–641 Mb, and BUSCO scores of ~94% (Table 1). Assembled chromosome sizes ranged from 16 to 712 Mb. More than 97% of the short reads aligned back to the respective chromosome‐level assembly, indicating high levels of completeness. The chromosome‐level assemblies were annotated with 17,040–18,033 protein‐coding genes (Table 1). The genome size of the race P25XY without genome assembly was estimated as 3.27 Gb by a k‐mer based approach (Figure S1).

**TABLE 1:** Genome assembly and annotation statistics for morabine grasshoppers.

Species/races	P24XY female	P24X0 male^a^
Genome size (Gb)	3.75	3.93
Scaffolds	15,849	30,905
N50 scaffold (Mb)	389	641
L50 scaffold count	4	3
Contigs	41,379	299,331
N50 contig (bp)	204,729	33,865
Protein‐coding genes	18,033	17,040
mRNA	20,240	19,076
BUSCO complete (%)	93.4	93.7
TE content (%)	70.25 (68.34^b^)	67.31 (61.05^b^)
*SINE* (*%*)	2.27 (2.77^b^)	3.23 (2.58^b^)
*LINE* (*%*)	25.08 (26.54^b^)	24.74 (24.76^b^)
*LTR* (*%*)	4.83 (4.10^b^)	3.13 (3.12^b^)
*DNA transposons* (*%*)	20.12 (17.41^b^)	16.73 (15.76^b^)
*RC* (*%*)	6.19 (5.98^b^)	4.91 (5.25^b^)
*Unknown* (*%*)	11.76 (11.54^b^)	14.57 (9.58^b^)
Satellite DNA (%)	0.19 (2.93^b^)	0.21 (7.43^b^)
Total repeat content %	70.44 (71.27^b^)	67.52 (68.48^b^)

^a^ Assembly stats post‐RaGOO (Alonge et al. 2019).

^b^ TE content estimated with dnaPipeTE (Goubert 2023; Goubert et al. 2015).

We characterized the repetitive DNA content in the chromosome‐level assemblies using homology‐based and *de novo* approaches. The total repeat content in the assembled genomes ranged from 67.52% to 70.44%, with long interspersed nuclear elements (LINEs) as the most common type of repeat (25.08%–26.72%) followed by DNA transposons (16.73%–20.12%) (Table 1). For the three races/species of the morabine with resequencing data, we annotated repeats based on short reads. The total repeat contents for these races/species were estimated to be 68.48%–75.94%. LINEs were the most common type of repeat (24.76%–28.46%) followed by DNA transposons (15.76%–19.77.12%). The satDNAs were poorly assembled/annotated in the chromosome‐level assemblies, but they represented 2.00%–7.43% of the resequencing reads (Table 1, Table S2).

#### 3.1.1 Identification of the Sex‐Linked Regions

**FIGURE 2:**
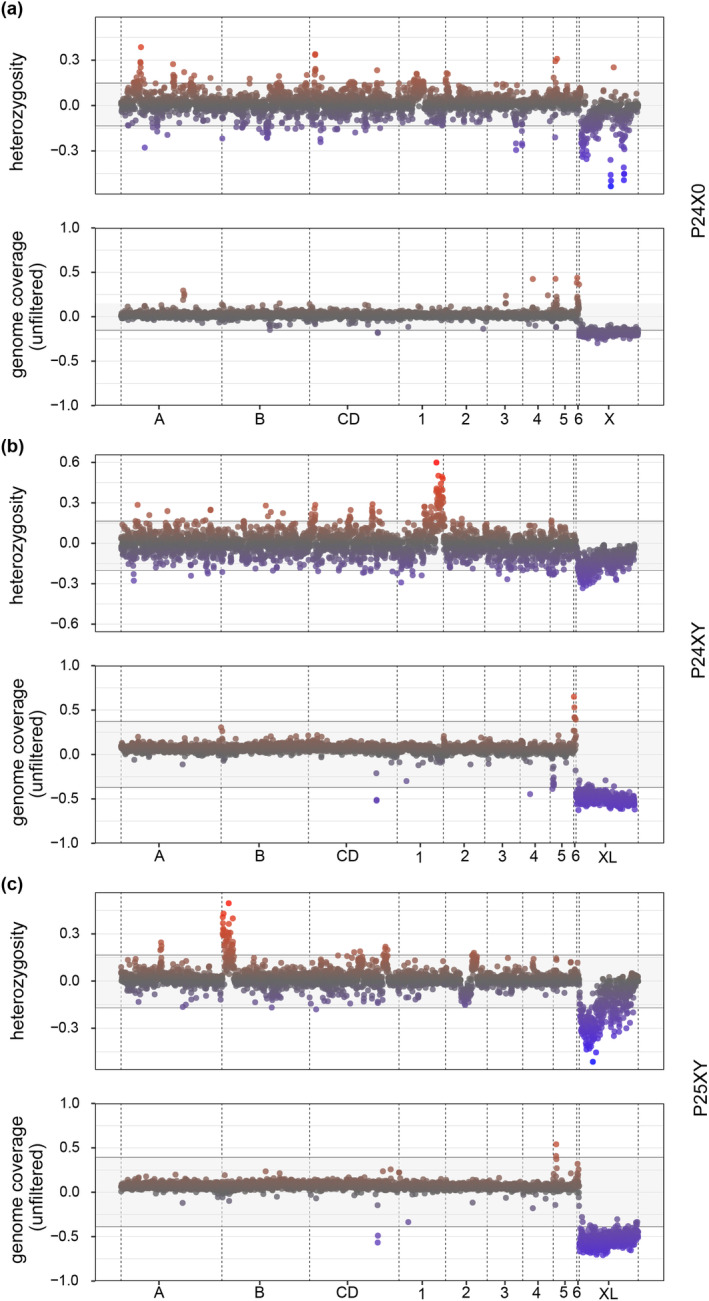
Genome‐wide sex differences in coverage and heterozygosity across 1 Mb windows plotted along chromosomes positions in the *Vandiemenella* morabine grasshopper genomes. (a) Chromosomal race P24X0, (b) P24XY, and (c) P25XY. Rows depict heterozygosity and genome coverage (unfiltered mismatch setting). The gray background indicates the 95% confidence intervals (CI), with data points exceeding these values marked in red (if higher) or blue (if lower). The data reveal sex‐linked regions encompassing chrX and/or XL across all races, as well as segments of chr1 (257–304 Mb) and chrB (0–75 Mb) in P24XY and P25XY morabine grasshoppers, respectively.

We identified the sex chromosomes based on read coverage and heterozygosity in resequencing reads from males and females (Figure 2). We reapplied mapping with different numbers of mismatches allowed, since differentiation between the sex chromosomes will create more mismatches between the differentiated neo‐Y chromosome and the reference (Figure S2). The fourth longest scaffold spanning 386–435 Mb in the assemblies showed pronounced sex differences in genome coverage compared to the rest of the chromosomes (Figure 2). The observed coverage patterns suggest that these scaffolds correspond to the male hemizygous X chromosome in P24X0 and the male hemizygous XL chromosome arms in P24XY and P25XY, consistent with karyotype descriptions. Genome alignments showed that the X chromosome (and/or XL) is homologous to the X chromosome of the desert locust *Schistocerca gregaria* (Figure S3), thereby confirming its homology with the X chromosome of other insects (Li, Mank, and Ban 2024; Toups and Vicoso 2023).

In P24XY, chr1 hosts a region of ~47.5 Mb (from 257 to 304.5 Mb) that shows sex differences in heterozygosity compared to the rest of the chromosomes. Likewise, in P25XY, a region on chrB, spanning 75 Mb (from 0 to 75 Mb), also shows pronounced sex differences in heterozygosity (Figure 2). The chr1 and chrB were involved in the X‐A centric fusion events that originated the neo‐sex chromosomes in P24XY and P25XY, respectively (see Figure 1). These areas with excess heterozygosity might therefore represent regions between neo‐X chromosome (XR arm) and neo‐Y chromosome that no longer undergo recombination (hereafter referred to as the sex‐linked regions). The female‐specific excess heterozygosity seen on the X or XL chromosome arms matches their hemizygosity in males, a condition where only one copy is present (Figure 2).

### 3.2 Gene Content of Neo‐Y Chromosomes

**FIGURE 3:**
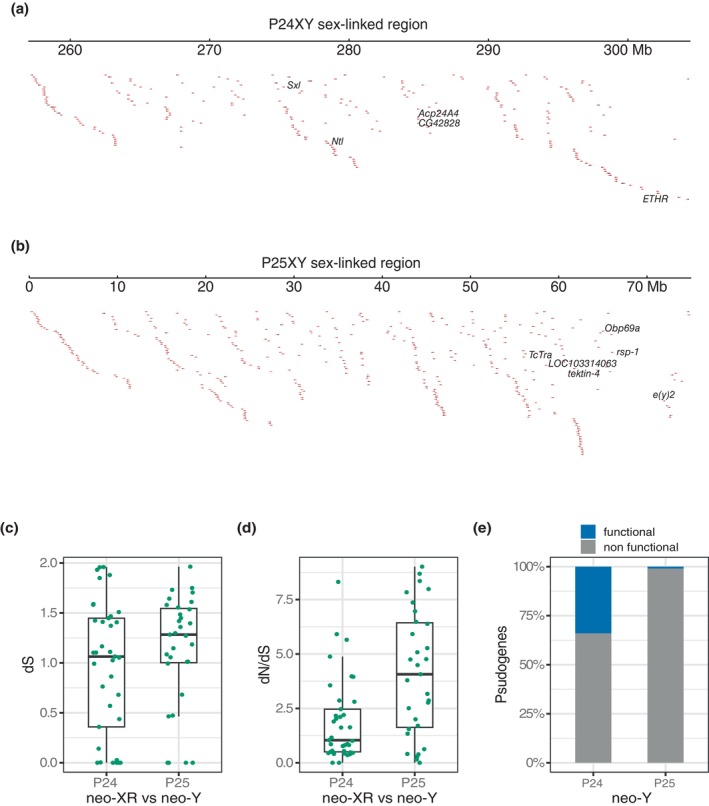
Tracks of genes and levels of divergence and degeneration in the sex‐linked regions of the *Vandiemenella* morabine grasshopper genomes. The sex‐linked regions in (a) P24XY and (b) P25XY span many loci indicated by triangles, some of which (highlighted) affect sex determination, gametogenesis, reproduction, fertility, sexual communication, and behavior. (c) Box plots show neo‐XR and neo‐Y synonymous site divergence estimates (dS values) for the sex‐linked regions in P24XY and P25XY. (d) Box plots shows the rate of non‐synonymous to synonymous substitutions (dN/dS values) for the neo‐XR and neo‐Y in the sex‐linked regions of P24XY and P25XY. The summary statistics in the boxplots represent the median (horizontal black bar), interquartile range (the thick white bar in the center), and the extreme of the distribution (thin black line). (e) Bar plot illustrating the levels of genetic degeneration (loss‐of‐function mutation) in P24XY and P25XY sex‐linked neo‐Y regions. The proportion of nonfunctional pseudogenes is used as proxy of the levels of gene degeneration in each sex‐linked region.

We predicted protein‐coding genes on the sex‐linked regions of the neo‐XRs P24XY and P25XY genomes that showed homology to proteins with known functions in *Drosophila melanogaster, Caenorhabditis elegans* and/or *Tribolium castaneum*. None of these genes were classified as pseudogenes. We identified 251 genes located in the sex‐linked neo‐XR region of the P24XY. Some were related to spermatogenesis, fertility, reproduction, and sexual dimorphism (Figure 3), which illustrates their integrated role as a putatively masculinizing supergene. These genes included sex lethal (*Sxl; Dmel\CG43770*) (Gramates et al. 2022), the neurotransmitter transporter‐like gene (*Ntl; Dmel\CG7075*) (Gramates et al. 2022), the accessory gland protein gene *24A4* (*Acp24A4*; *Dmel\CG31779*) (Gramates et al. 2022), the serine‐type endopeptidase inhibitor activity (*Dmel\CG42828*) expressed in testis (Gramates et al. 2022), and the *ETHR* essential in initiating the molt‐related behaviors of ecdysis, which in adults function to promote juvenile hormone signaling and reproduction (Gramates et al. 2022). Paralogs of *Sxl* were located on chrCD and chr4, and a paralog of *Ntl* was also present in chr2. In P24X0, *Sxl* was found on chr4, but it was missing from the unfused chr1.

We identified 470 genes situated within the sex‐linked neo‐XR region of P25XY (chrB) (Figure 3b). Among the annotated genes was the pre‐mRNA‐splicing regulator female‐lethal(2)D (Transformer, *TcTra, LOC663936*), crucial for female sex determination by regulating the sex‐specific splicing of *dsx* pre‐mRNA (Sánchez 2008). This gene was found on the unfused chrB of P24X0, found on the P24XY neo‐Y chromosome, but lies outside the sex‐linked neo‐Y region. We found the presence of odorant receptor 4 (*LOC103314063*) and general odorant‐binding protein 69a (*Obp69a, LOC656765*), which are key genes involved in insect sexual communication and behavior, including feeding, mating, and egg‐laying (Bentzur et al. 2018; S. Zhang et al. 2023). A paralog of *LOC103314063* was also detected in chr2. Moreover, we identified the *tektin‐4* (*LOC661941*), known to play essential roles in the mechanics of sperm motility across evolutionarily diverse species (Roy et al. 2007). We found the gene *rsp‐1* (*CELE_W02B12.3*), which serves a functionally redundant role in spermatogenesis and growth rate control (Kawano, Fujita, and Sakamoto 2000; Longman, Johnstone, and Cáceres 2000). Furthermore, we observed the presence of the testis‐specific paralog of the ubiquitously expressed transcription and mRNA export factor e(y)2 (Dmel\CG14612).

### 3.3 Age and Genetic Degeneration of the Neo‐Y Chromosomes

We used sequence divergence in protein‐coding genes to estimate the relative divergence time of the sex‐linked region of the two neo‐XY chromosomal races. Sequence divergence between the individual neo‐XR and neo‐Y gametologous gene pairs was calculated by synonymous nucleotide divergence (dS). We phased, clustered, aligned, and filtered neo‐Y and neo‐XR gametolog sequences from multiple males and females, which resulted in 38 neo‐XY gene pairs within the sex‐linked region in the P24XY genome, and 31 neo‐XY gene pairs within the sex‐linked region in P25XY. The sex‐linked regions showed different levels of divergence, consistent with different ages. In P24XY, the median dS value for the sex‐linked region was 1.06, while in P25XY, it was 1.2 (Figure 3c). We detected significant variations in dS values between the sex‐linked regions (independent *t*‐test; *t* = −5.97, *df* = 32.72, *p* = 1.068e‐06). In P24XY, the median dN/dS value for the sex‐linked region was 1.04, whereas in P25XY, it was 4.07 (Figure 3d). Additionally, these rates showed significant differences between the sex‐linked regions (independent *t*‐test; *t* = −3.66, *df* = 50.46, *p* = 5.9e‐04).

To further quantify the levels of genetic degeneration in each sex‐linked region, we classified neo‐XR and neo‐Y pseudogene sequences as functional or nonfunctional, based on the presence of intact open reading frames (ORFs). The estimated proportion of pseudogene sequences without intact ORFs was then used as a measure of degeneration. The P24XY sex‐linked neo‐Y region showed 73 pseudogenes, with 25 having ORFs and 48 lacking ORFs, respectively. In contrast, the P25XY sex‐linked neo‐Y region featured 137 pseudogenes, with only one having an ORF and 136 lacking ORFs (Figure 3e).

### 3.4 Repeat Accumulation

**FIGURE 4:**
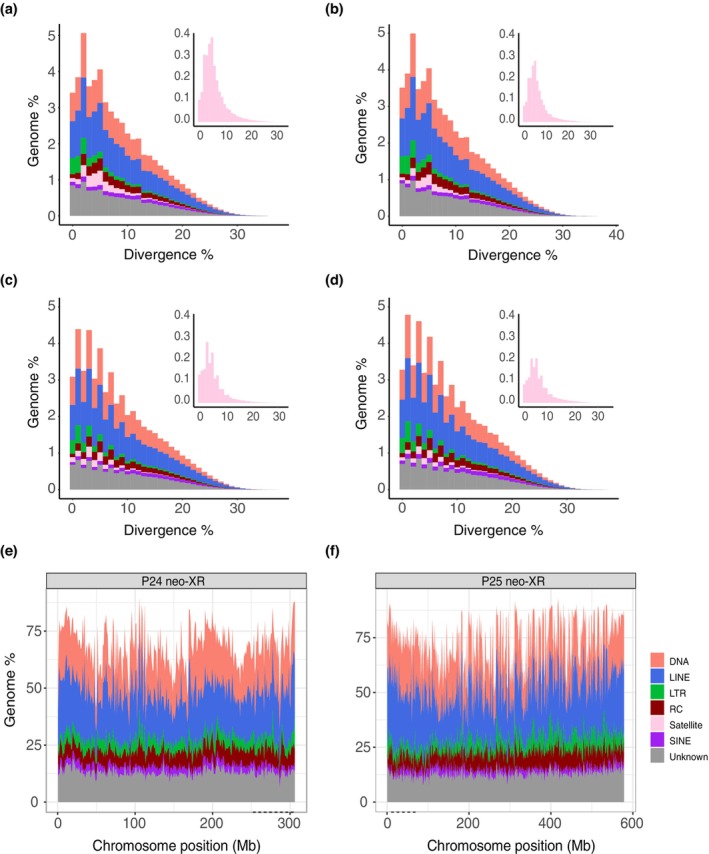
Repeat landscapes across *Vandiemenella* morabine grasshopper genomes. (a‐d) Read‐based repeat content comparison in (a) male versus (b) female of P24XY, and (c) male versus (d) female of P25XY. The inserts depict the satDNA landscapes in each genome. The histograms show the distribution of the BLASTn divergence analysis to the consensus on the *x* axis and the satDNA abundance (as % of the reads) on the *y* axis for males and females. Each bin on the *x* axis represents 1% divergence. (e, f) Assembly‐based repeat content along the neo‐XR chromosomes of P24XY and P25XY. Percentage of repeat‐derived base pairs shown per window of 1 Mb along the neo‐XR chromosomes of P24XY and P25XY, following the same color scheme as (a–d). The alternating black dashed lines below the *x* axis mark the sex‐linked regions (evolutionary strata) discussed in the main text.

We tested for sex differences in TE accumulation in the sex‐linked regions. TE accumulation in the P24XY genome shows a slight difference between males (67.23%) and females (68.58%). Similarly, in the P25XY genome, males (73.25%) have a slightly larger TE accumulation compared to females (72.83%) (Figure 4a–d, Table S1). The P24XY sex‐linked region (257–304 Mb) with more recent histories of recombination suppression had a slightly larger median TE density (61.91%) across 1 Mb windows than the rest of the chromosome (60.41%) (Figure 4e, Figure S4). The P25XY sex‐linked region (0–75 Mb), however, had a median TE density (64.1%) that was smaller than the rest of the chromosome (68.33%) (Figure 4f, Figure S4). The differences in TE density on the sex chromosomes did not statistically differ from other regions of the genome (Mann–Whitney U test with continuity correction; *p* > 0.1). SatDNAs, however, were more abundant (up to 1.6 times) in male than in female genomes (Figure 4a,b; Table S2).

### 3.5 Chromosomal Rearrangements in Neo‐Sex Chromosomes

**FIGURE 5:**
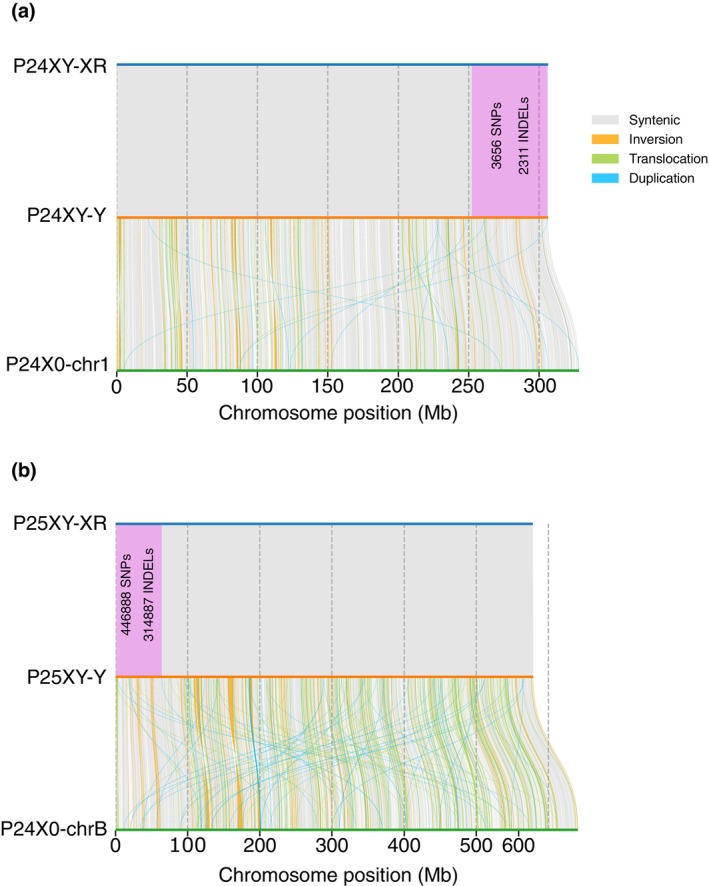
Chromosomal rearrangements between the neo‐sex chromosomes and their ancestral counterparts in *Vandiemenella* morabine grasshoppers. **(a)** Visualization of syntenic regions and intrachromosomal rearrangements between P24XY neo‐XR and neo‐Y, as well as between neo‐Y and its ancestral counterpart P24X0 chr1. **(b)** Visualization of syntenic regions and intrachromosomal rearrangements between P25XY neo‐XR and neo‐Y, along with between neo‐Y and its ancestral counterpart P24X0 chrB. The pink shaded area highlights the cumulative count of SNPs and indels within the sex‐linked regions of P24XY and P25XY.

The karyotypic differences among morabine grasshoppers (Figure 1a) suggest the occurrence of multiple chromosome rearrangements that come in many forms (inversions, translocations, centric fusions, and fissions, deletions, and insertions). We thus mapped chromosome rearrangements by performing pair‐wise whole‐genome alignments of the P24XY and P25XY neo‐sex chromosomes together with their ancestral counterparts in P24X0, that is, chr1 and chrB, respectively (Figure 5). Following the alignment of whole genomes (with 95% identity) and the removal of short alignments (< 2000 bp), we showed that the sex‐linked regions evolved by the accumulation of SNPs and insertions/deletions (indels). We identified a substantial number of these variations: 3,656 SNPs (median 51 SNPs per Mb) and 2,311 indels (median 40 indels per Mb) within the P24XY sex‐linked region (Figure 5a) and a much larger number of 446,888 SNPs (median 703 SNPs per Mb) and 314,887 indels (median 564 indels per Mb) in the P25XY sex‐linked region (Figure 5b). The parts of these neo‐sex chromosomes that still recombine share a similar gene order (Figure 5a,b).

The examination of chromosome rearrangements between the P24XY neo‐Y chromosome and its ancestral counterpart, P24X0 chr1, revealed 69 inversions (mean size 27.42 kb/Mb), 1909 translocations (mean size 13.84 kb/Mb), and 791 duplications (mean size 52.18 kb/Mb). A larger number of chromosome rearrangements were observed between the P25XY neo‐Y chromosome and P24X0 chrB, including 179 inversions (mean size 48.57 kb/Mb), 7061 translocations (mean size 26.64 kb/Mb), and 4859 duplications (mean size 16.97 kb/Mb) (Figure 5). Interestingly, these chromosome rearrangements were distributed randomly across the chromosomes (Figure 5a,b).

## 4 Discussion

Independent X‐A chromosome fusions have given rise to three chromosomal races with neo‐XY sex‐determining systems in the *Vandiemenella viatica* species group (White 1978). Using newly generated chromosome‐level assemblies, annotations, and resequencing data, we identified chromosomes in both fused (neo‐XY) and unfused (X0) states in the two chromosomal races. Our data show that segments of the neo‐X/neo‐Y have ceased to recombine and thus became sex‐linked in both P24XY and P25XY. The sex‐linked region is larger and older in P25XY than in P24XY, as indicated by greater level of divergence between neo‐XR and neo‐Y gametologs in P25XY. Furthermore, we show that both fused autosomes harbor different sex‐specific genes involved in spermatogenesis, fertility, and reproduction that are now tightly linked to the putatively sex‐determining loci (*Sxl, TcTra*). Some of the genes with sex‐specific functions have paralogs on other autosomes, indicating that duplication/translocation has happened, possibly after the fusion. We found an enrichment of point mutations and structural variants on the neo‐Y gametologs, but, perhaps surprisingly, no enrichment of transposable elements (TEs). These results shed new light on the early evolution of sex chromosomes.

One central question is whether the fusions spread with the aid of natural selection. Selection might favor chromosomal fusions if the fusions captured alleles that profit from being transmitted through the sex they benefit (Ponnikas et al. 2018). Alternatively, the fusion might have spread through non‐selective processes, and the identity of the captured chromosome might be random with respect to selection. The fact that neo‐sex chromosomes in the *viatica* group have evolved at least three times with different autosomes does not indicate that chromosomal identity plays a pivotal role. Furthermore, we found no overlap in the set of genes with putative male‐specific function within the sex‐linked region of P24XY and P25XY. However, both neo‐Y chromosomes host sex‐specific genes that could potentially undergo positive selection if they become sex linked, even if the lack of overlap in gene content suggests that any effects are idiosyncratic. While we cannot entirely rule out a role of chromosomal identity, there is no strong evidence to suggest that particular autosomes were predisposed to become neo‐sex chromosomes. The identity of neo‐sex chromosomes rather appears to be random, and such randomness might have contributed to the diversity of genomes observed across different lineages in the *viatica* species group and possibly to speciation itself.

The second intriguing question is when and why recombination ceases after the formation of neo‐sex chromosomes. Our data show that recombination can cease after relatively short time spans (< 3.1 Myr), assuming that species divergence is linked to or driven by sex chromosome diversification. However, the cessation of recombination did not immediately affect the entire chromosome; rather, it seems to progress gradually. Reduced recombination between neo‐XR and neo‐Y gametolog gene pairs may be a consequence of selection favoring linkage between sexually antagonistic alleles and the sex‐determining region (Ponnikas et al. 2018; Rice 1984; Wright et al. 2016). Nonmutually exclusive proximate causes for reduced recombination include sequence divergence (Charlesworth 2021a), structural variation (Ponnikas et al. 2018; Wright et al. 2016), accumulation of repetitive DNA and heterochromatinization (Ponnikas et al. 2018; Wright et al. 2016), or a spatial effect of the chromosomal fusion leaving no space for crossing over because of the bulky X chromosome (Castillo, Marti, and Bidau 2010).

Sequence divergence has been shown to reduce recombination owing to impaired sequence alignment during meiosis (Charlesworth 2021a, 2021b), and indeed sequence divergence and sex differences in heterozygosity were large between gene pairs located on neo‐XR and neo‐Y of P24XY and P25XY. While it is possible that sequence divergence occurred after recombination had already been reduced, sequence divergence might produce positive feedback loops that allow the non‐recombining region to expand gradually. Previous cytological studies in morabine grasshoppers demonstrate that the X‐A fusion affects meiotic recombination in the neo‐XY of males. The proximal chiasma of the neo‐Y chromosomes has shifted toward the interstitial position in P25XY or the distal position in P24XY, thereby creating recombination‐free regions (White 1978; White, Blackithr, and Cheney 1967). The relocation of chiasmata thus likely contributes to reduced recombination. There is no cytological evidence that the neo‐Y chromosomes have been heterochromatinized in morabine grasshoppers, so this is unlikely to be a significant factor. We did not find evidence of major chromosome rearrangements, inversions in particular, that might span the sex‐linked region and would have contributed to reduced recombination. Similarly, we did not find accumulation of repetitive DNA, also making repetitive DNA an unlikely driver. We believe that reduced recombination likely occurred through a combination of relocation of the chiasmata and sequence divergence via accumulation of SNPs and indels. This interpretation aligns with the idea of soft boundaries and gradual expansion (Natri, Shikano, and Merilä 2013; Sigeman et al. 2021) as seen in the difference between the P24XY and P25XY lineages, contrasting with discrete evolutionary strata found in old sex chromosomes like mammals (Lahn and Page 1999; Skaletsky et al. 2003) and birds (Handley, Ceplitis, and Ellegren 2004; Yazdi, Silva, and Suh 2020; Zhou et al. 2014).

The third intriguing question is if post‐fusion translocations have contributed to an accumulation of sex‐specific and sexually antagonistic genes on the neo‐sex chromosomes. The consequences of X‐autosome fusions can vary widely. In some cases, neo‐sex chromosomes can shuffle genes associated with reproductive isolation and speciation (Payseur, Presgraves, and Filatov 2018; Wang et al. 2022), while in others, they may have little effect on fitness (Kirkpatrick and Barton 2006). If the establishment of recombination‐free regions strongly favor the accumulation of more sexually antagonistic alleles, selection may favor reduced recombination to retain them in the appropriate sex, eventually allowing variability to be released as seen in the P24XY and P25XY sex‐linked regions. We did find paralogs of some of the sex‐specific genes on neo‐Y now on other autosomes. These paralogs seem to be intact and thus suggest that duplications have happened. Many of these genes were apparently copied to the neo‐Ys and sex‐specific genes were over‐represented on neo‐Ys as compared to their ancestral counterparts. Whether duplications have happened before or after the fusion cannot be determined, but it seems possible that they happened following the fusion and represent the first step toward translocation of male‐specific genes from autosomes to neo‐Y.

The fourth important question is how fast neo‐sex chromosomes degenerate. Degeneration in sex chromosomes can manifest as loss‐of‐function mutation of Y‐linked genes, pseudogenization, and the accumulation of repetitive elements in the sex‐linked regions (Charlesworth 2021a). Both neo‐Y sex‐linked regions in *Vandiemenella* grasshoppers have degenerated, as evidenced by sequence divergence and pseudogenization, like the sex chromosomes in mammals (Lahn and Page 1999; Skaletsky et al. 2003), birds (Handley, Ceplitis, and Ellegren 2004; Yazdi, Silva, and Suh 2020; Zhou et al. 2014), and *Drosophila* (Bachtrog 2013). This indicates fast degeneration of the young neo‐sex chromosomes of morabine grasshoppers following the reduction of recombination. Morabine grasshopper genomes host a large fraction of repeats that contribute up to ~80% to DNA content, owing to the recent proliferation of distinct classes of TEs (Palacios‐Gimenez et al. 2020). Surprisingly, we did not find elevated densities of TEs in the sex‐linked regions, which contrast with other grasshoppers and crickets with neo‐sex chromosomes showing differential accumulation of repeats between sexes (Ferretti et al. 2020; Palacios‐Gimenez et al. 2017, 2018). We therefore suspect that the timing of X‐A fusion and the cessation of recombination did not match with the timing of TE activity. In particular, if TE spread occurs in bursts, this may be quite unlikely for the early stages of sex chromosome evolution. Indeed, there is evidence from other grasshoppers that TE activity occurred in bursts at certain points in the past (Shah, Hoffman, and Schielzeth 2020). While the data suggest that TE enrichment does not necessarily occur over short time‐frames, theory leads us to anticipate a disproportional accumulation of TEs in the long run (Charlesworth 2021a; Ponnikas et al. 2018).

A fifth important question is whether the chromosomal fusion and emergence of a new sex‐linked region also promote further structural rearrangements and ultimately fuel periods of genomic fluidity. While we did not find a major chromosomal inversion that covers the entire sex‐linked region, we found genomic differences ranging from SNPs and indels within the sex‐linked regions as well as complex chromosome rearrangements outside the sex‐linked regions. These chromosome rearrangements had remained undetectable by previous cytological analysis (White 1978; White, Blackithr, and Cheney 1967). The large repeat content might have provided the source for ectopic recombination to work, giving rise to major chromosome rearrangements like those in many other taxa (Huang et al. 2022; Montgomery et al. 1991). Chromosome rearrangements may be an evolutionary cause of the differences in gene content across morabine grasshoppers (spanned 17,040–18,033 protein‐coding genes), with gain of genes in some lineages and losses in others. While we cannot benchmark the frequency of structural variants, it seems possible that the X‐A fusion promotes further rearrangements. Interestingly, however, inversions did not yet happen within the sex‐linked regions.

We annotated protein‐coding genes within the non‐recombining regions of the newly evolved neo‐XY sex chromosomes. Several genes of interest in the sex‐linked regions contribute to the sex‐determining pathway, spermatogenesis, fertility, and reproduction, suggesting an integral role of the neo‐Y as a putatively masculinizing supergene. However, we cannot determine which of these genes are the master sex‐determining genes in morabine grasshoppers; functional assessment of these candidate genes is thus needed. Surprisingly, there was hardly overlap between these genes on the neo‐Y chromosomes of P24XY and P25XY. Some of the genes apparently underwent duplications, as evidenced by the occurrence of paralogs in different morabine grasshoppers. Two notable instances include the incorporation of sexual differentiation regulatory genes *Sxl* and *TcTra*, which occurred independently through fusions into novel sex‐limited environments in P24XY and P25XY. Another notable gene in the P24XY neo‐Y with test‐specific function in *D. melanogaster* is *Ntl* that induces male sterility by significantly reducing glycylated tubulin production (Gramates et al. 2022). Parental or duplicated *Sxl* and *Ntl* also existed as autosomal copies, indicating that neo‐Ys underwent not only fusion but also post‐fusion gene duplication/translocation.

Our analysis of newly generated chromosome‐level assemblies of replicated X‐A fusions provides several new insights on sex chromosome evolution. The patterns we identify suggest the following evolutionary history of neo‐sex chromosomes in the *viatica* species group: (i) The initial chromosomal fusion most likely occurred with a random chromosome that hosted male‐specific genes, and the increase in frequency of the X‐A fusion most likely resulted from non‐selective processes. (ii) Recombination was reduced in parts of the neo‐sex chromosomes, initially most likely through shifting of the chiasmata and further fueled by sequence divergence among the gametologs. (iii) The region of reduced recombination gradually expanded through further accumulation of sequence divergence and positive feedback loops. Notably, we found no signs of major structural variants contributing to a reduction in recombination. (iv) The neo‐Y chromosome began to degenerate, as is evidenced in particular by pseudogenization. Surprisingly, we found no evidence of significant repeat accumulation in the non‐recombining region of neo‐Ys, most likely due to a period of low TE activity following the X‐A fusion. (v) Some translocations of genes with male‐specific function to neo‐Ys seem to have happened, although we cannot unambiguously demonstrate that these have happened after the fusion.

## Author Contributions

Octavio M. Palacios‐Gimenez contributed to the conceptualization. Suvratha Jayaprasad, Simon J. Ellerstrand, Valentina Peona, Roberto Rossini, Ignas Bunikis, Remi‐André Olsen, and Octavio M. Palacios‐Gimenez contributed to the formal analysis; Octavio M. Palacios‐Gimenez, Holger Schielzeth, Takeshi Kawakami, and Alexander Suh contributed to the funding acquisition. Suvratha Jayaprasad, Valentina Peona, and Octavio M. Palacios‐Gimenez contributed to the investigation; Takeshi Kawakami, Alexander Suh, and Octavio M. Palacios‐Gimenez contributed to the project administration; Octavio M. Palacios‐Gimenez, Holger Schielzeth, Takeshi Kawakami, Alexander Suh, Olga V. Pettersson, Carl‐Johan Rubin, Elisabet Einarsdottir, Franziska Bonath, Tessa M. Bradford, and Steven J. B. Cooper contributed to the resources; Octavio M. Palacios‐Gimenez and Holger Schielzeth contributed to the supervision. Suvratha Jayaprasad and Octavio M. Palacios‐Gimenez contributed to the validation. Suvratha Jayaprasad and Octavio M. Palacios‐Gimenez contributed to the visualization; Octavio M. Palacios‐Gimenez and Holger Schielzeth contributed to the writing of the original draft; Octavio M. Palacios‐Gimenez, Holger Schielzeth, Takeshi Kawakami, Alexander Suh, Suvratha Jayaprasad, Valentina Peona, Steven J. B. Cooper, Elisabet Einarsdottir, Simon J. Ellerstrand, and Bengt Hansson contributed to the writing of the remaining drafts. All authors read and approved the final manuscript.

## Conflicts of Interest

The authors declare no conflicts of interest.

## Supporting information


Data S1.

## Data Availability

The assemblies and raw sequencing data are available under the NCBI accession and listed in the Table S1. All study data are included in the article and/or Supporting Information. The software and codes used in this study are publicly available, with corresponding versions indicated in Materials and Methods. The PhaseWY pipeline is still under development, and the code used for this study will be published following publication of the pipeline.
