# Supplementary material for: Orthopteran Neo‐Sex Chromosomes Reveal Dynamics of Recombination Suppression and Evolution of Supergenes
Source: Mol Ecol. 2024 Oct 30;33(23):e17567. doi: 10.1111/mec.17567 (PMC11589690; doi:10.1111/mec.17567)
Supplement: Supplementary file 1 — Data S1. [file MEC-33-e17567-s001.pdf]

MOLECULAR ECOLOGY

Supplemental Information for:

Orthopteran neo-sex chromosomes reveals dynamics of recombination suppression and evolution of supergenes

Suvratha Jayaprasad[a], Valentina Peona[b,†], Simon J. Ellerstrand[c], Roberto Rossini[c,d], Ignas Bunikis[e], Olga V. Pettersson[e], Remi-André Olsen[f], Carl-Johan Rubin[g], Elisabet Einarsdottir[h], , Franziska Bonath[h], Tessa M. Bradford[i,j], Steven J. B. Cooper[i,j], Bengt Hansson[c], Alexander Suh[c,k,‡], Takeshi Kawakami[l,], Holger Schielzeth[a,m], Octavio M. Palacios-Gimenez[a,b,m,*]

Jena DE-07743, Germany. [b]Department of Organismal Biology – Systematic Biology, Evolutionary Biology Centre, Uppsala University, Uppsala SE-75236, Sweden. [c]Department of Biology, Lund University, Lund SE-22363, Sweden. [d]Department of Biosciences, University of Oslo, Oslo NO-0316. [e]Uppsala Genome Center, Department of Immunology, Genetics and Pathology, Uppsala University, National Genomics Infrastructure hosted by SciLifeLab; Uppsala SE-75123, Sweden. [f]Science for Life Laboratory, Department of Biochemistry and Biophysics, Stockholm University, Solna SE-17165, Sweden. [g]Department of Medical Biochemistry and Microbiology – Disciplinary Domain of Medicine and Pharmacy, Faculty of Medicine, Uppsala University, Uppsala SE-75123, Sweden. [h]Science for Life Laboratory, Department of Gene Technology, KTH-Royal Institute of Technology, SE-17121 Solna, Sweden. [i]Evolutionary Biology Unit, South Australian Museum, Adelaide, SA 5000, Australia. [j]School of Biological Sciences and Environment Institute, The University of Adelaide, Adelaide, SA 5005, Australia. [k]School of Biological Sciences, University of East Anglia, Norwich Research Park, Norwich NR4 7TU, UK. [l]Embark Veterinary, Inc., Boston, MA 02210, USA, [m]German Centre for Integrative Biodiversity Research (iDiv) Halle-Jena-Leipzig, Puschstraße 4, 04103 Leipzig, Germany.

[†]Present address: Swiss Ornithological Institute, Sempach, Switzerland.

[‡]Present address: Centre for Molecular Biodiversity Research, Leibniz Institute for the Analysis of Biodiversity Change, Zoologisches Forschungsmuseum A. Koenig, Adenauerallee 160, DE-53113 Bonn, Germany.*octavio.palacios@ebc.uu.se

Table of Contents:

**Supplementary Figure 1**	Page 2
**Supplementary Figure 2**	Page 2-3
**Supplementary Figure 3**	Page 4
**Supplementary Figure 4**	Page 5
**Supplementary Table 1**	Page 6
**Supplementary Table 2**	Page 7
**Legend for Dataset 1**	Page 7
**SI References**	Page 7-8

MOLECULAR ECOLOGY

Supplementary Figure 1. K-mer coverage profiles from GenomeScope (Ranallo-Benavidez et al., 2020) showing the estimate overall characteristics of P25XY morabine grasshopper genome, including genome size, heterozygosity rate and repeat content from unprocessed short reads. Profiles were measured using k = 21.

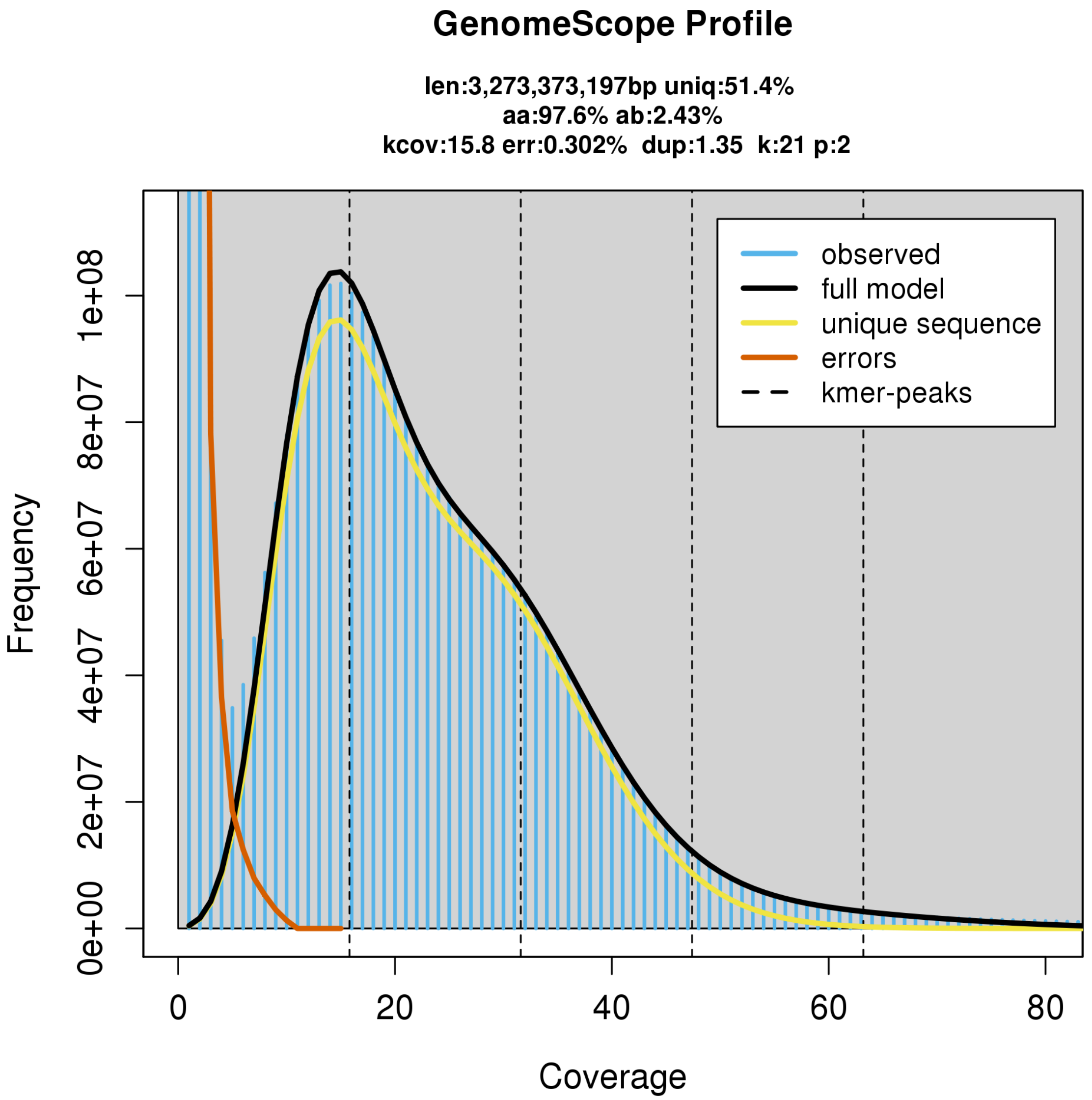

Supplementary Figure 2. Genome-wide sex differences in coverage and heterozygosity across 1 Mb windows plotted along chromosomes positions in the Vandiemenella morabine grasshopper genomes. **(a)** Chromosomal race P24X0, **(b)** P24XY, and **(c)** P25XY. Rows depict heterozygosity and genome coverage under three conditions: strict filtering (0 mismatches allowed, 0 mms), intermediate filtering (≤ 2 mismatches, ≤ 2 mms), and no filtering (unfiltered). The grey background indicates the 95% confidence intervals (CI), with data points exceeding these values marked in red (if higher) or blue (if lower). The data reveal sex-linked regions encompassing chr X and/or XL across all races, as well as segments of chr1 (257-304 Mb) and chrB (0-75 Mb) in P24XY and P25XY morabine grasshoppers, respectively.

MOLECULAR ECOLOGY

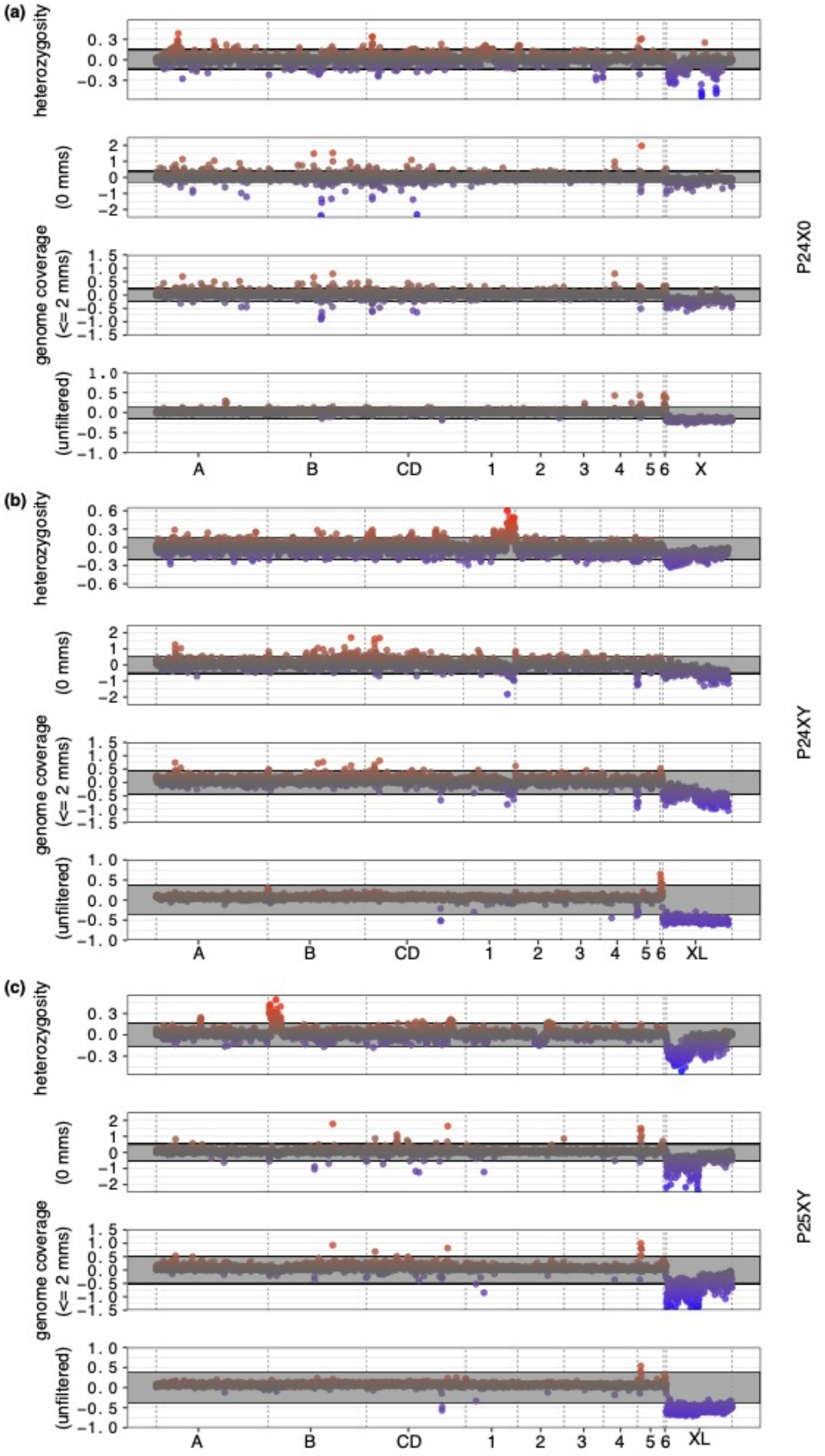

MOLECULAR ECOLOGY

Supplementary Figure 3. The identified collinear anchors between the desert locust *Schistocerca greagria* assembly (Sgreg) and the morabine grasshopper P24XY. Each dot is plotted based on the start coordinate of the *S. gregaria* and query genome P24XY of each anchor. Collinear anchors on the same strand between the *S. gregaria* genome and query genome P24XY are shown in blue, otherwise red. The alignment and plot were generate with AnchorWave (Song et al., 2022).

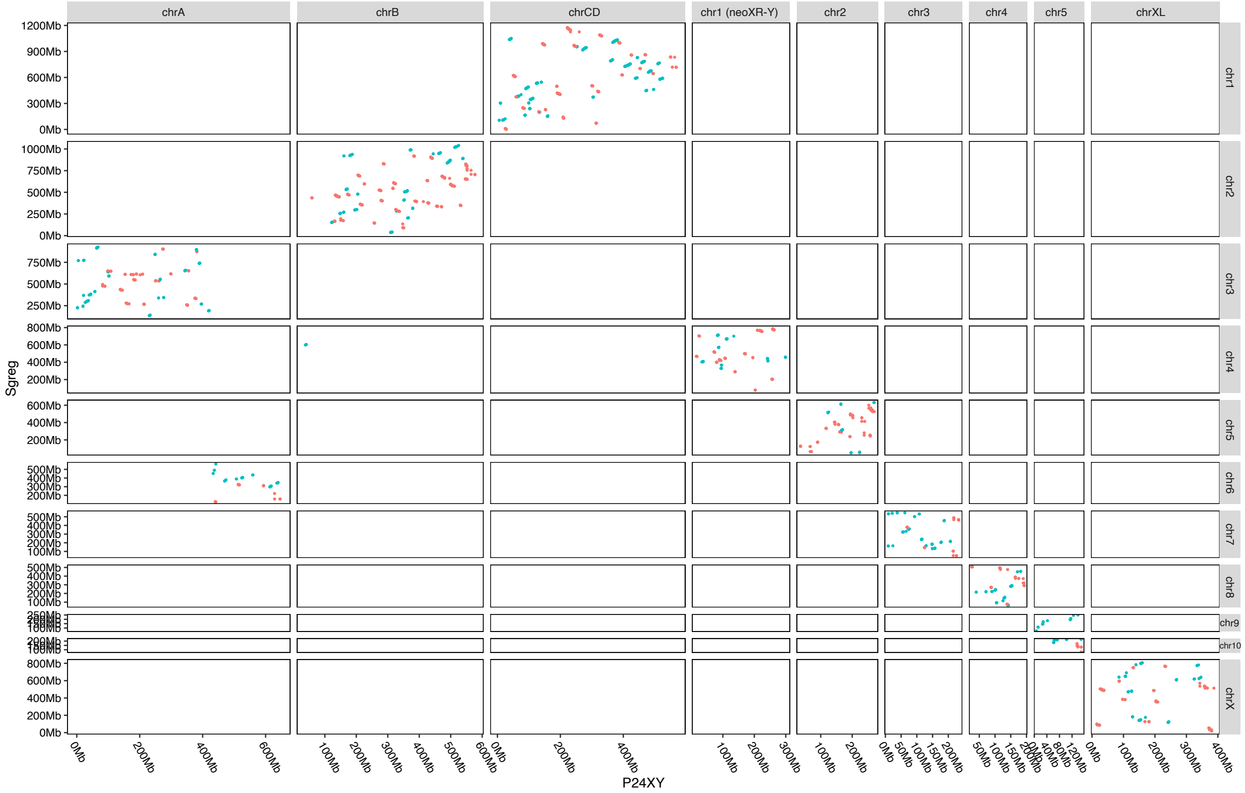

MOLECULAR ECOLOGY

Supplementary Figure 4. Assembly-based repeat landscape in the Vandiemenella morabine grasshopper genomes. **(a-c)** P24X0 and **(b-d)** P24XY. **(a-b)** Percentages of repeat elements for the entire autosomal assembly (A), the arms of the neo-X chromosome referred to as chrXL (the arm derived from the original X chromosome), and XR, the arm which share homology with the neo-Y. The ancestral unfused chr1 in P24X0 race corresponds to the neo-XR(Y) in P24XY. **(c-d)** Comparison of the repeat content across autosomes (chrA as a representative) and the unfused chr1, chrX(L) and the neo-XR sex chromosomes. Percentage of repeat-derived base pairs shown per window of 1 Mb, following the same color scheme as **(b-d)**.

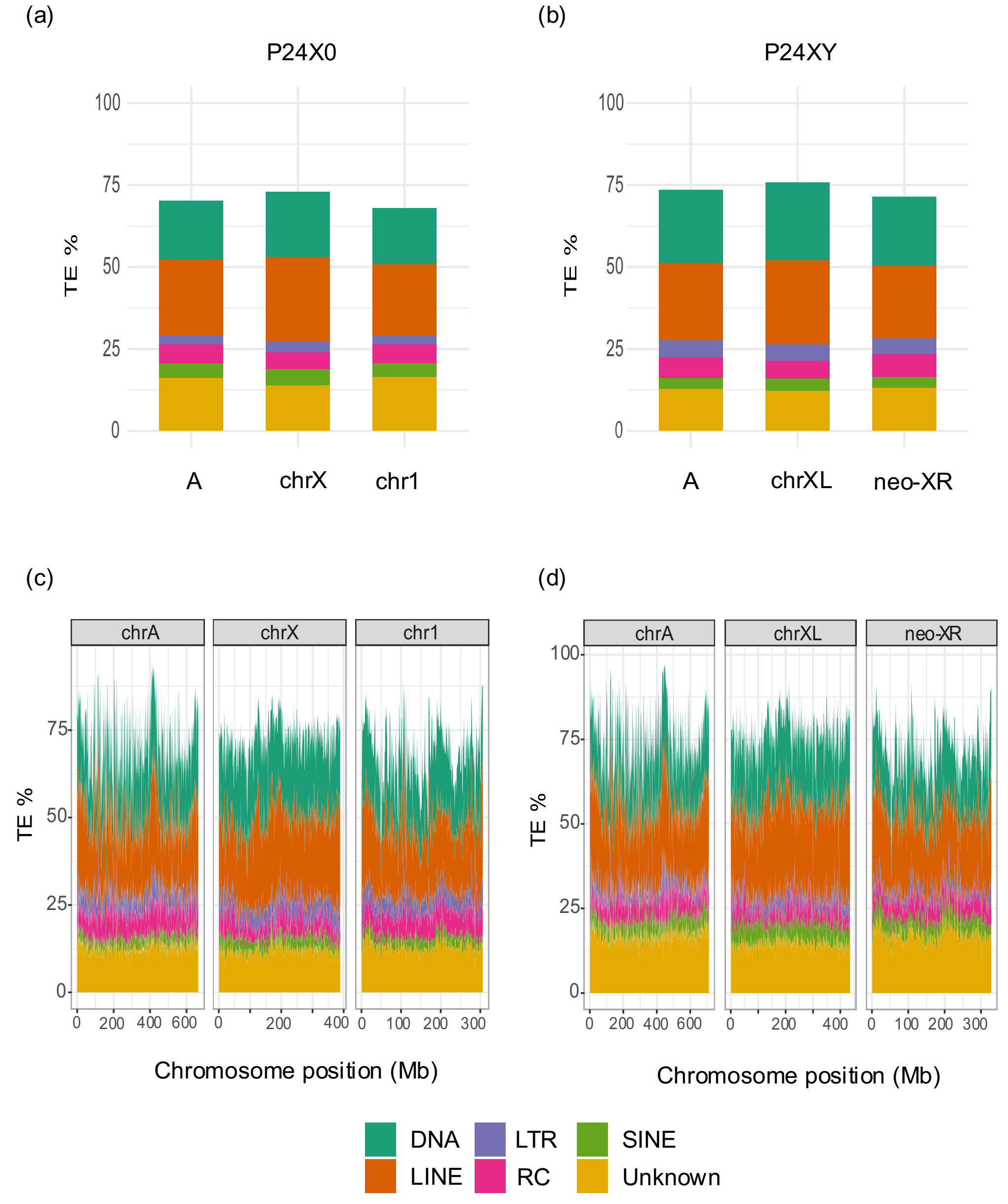

MOLECULAR ECOLOGY

Supplementary Table 1. Sequencing data from SRA or this study

Accession	Sequencing type	Sample	Source
PRJNA945230	PacBio	P24XY female head	This study
PRJNA945230	Hi-C	P24XY female carcass	This study
PRJNA945230	Hi-C	P24XY female carcass	This study
PRJNA945230	10X Genomics Chromium linked-read	P24XY female head	This study
PRJNA945230	resequencing	P24X0 male head	This study
PRJNA945230	resequencing	P24X0 male head	This study
PRJNA945230	resequencing	P24X0 male head	This study
PRJNA945230	resequencing	P24X0 female head	This study
PRJNA945230	resequencing	P24X0 female head	This study
PRJNA945230	resequencing	P24X0 female head	This study
PRJNA945230	resequencing	P24X0 female head	This study
PRJNA945230	resequencing	P24XY male head	This study
PRJNA945230	resequencing	P24XY male head	This study
PRJNA945230	resequencing	P24XY male head	This study
PRJNA945230	resequencing	P24XY female head	This study
PRJNA945230	resequencing	P24XY female head	This study
PRJNA945230	resequencing	P24XY female head	This study
PRJNA945230	resequencing	P25XY male head	This study
PRJNA668746	10X Genomics Chromium linked-read	P24X0 male head	(Palacios-Gimenez et al., 2020)
PRJNA668746	10X Genomics Chromium linked-read	P24XY male head	(Palacios-Gimenez et al., 2020)
GCA_019457785.1	10X Genomics Chromium linked-read	P24X0 male assembly	(Palacios-Gimenez et al., 2020)
PRJNA668746	RNA-seq	P24X0 male testis	(Palacios-Gimenez et al., 2020)
PRJNA668746	RNA-seq	P24X0 male head	(Palacios-Gimenez et al., 2020)
PRJNA668746	RNA-seq	P24X0 male leg	(Palacios-Gimenez et al., 2020)
PRJNA668746	RNA-seq	P24X0 female ovary	(Palacios-Gimenez et al., 2020)
PRJNA668746	RNA-seq	P24X0 female head	(Palacios-Gimenez et al., 2020)
PRJNA668746	RNA-seq	P24X0 female leg	(Palacios-Gimenez et al., 2020)
PRJNA668746	RNA-seq	P24XY male testis	(Palacios-Gimenez et al., 2020)
PRJNA668746	RNA-seq	P24XY male head	(Palacios-Gimenez et al., 2020)
PRJNA668746	RNA-seq	P24XY male leg	(Palacios-Gimenez et al., 2020)
PRJNA668746	RNA-seq	P24XY female ovary	(Palacios-Gimenez et al., 2020)
PRJNA668746	RNA-seq	P24XY female head	(Palacios-Gimenez et al., 2020)
PRJNA668746	RNA-seq	P24XY female leg	(Palacios-Gimenez et al., 2020)

MOLECULAR ECOLOGY

Supplementary Table 2. Read-based quantification of repeats across morabine grasshoppers using 0.1x genome coverage per sample. Density of different classes of repeats estimated by dnaPipeTE (Goubert, 2023; Goubert et al., 2015) using our generated custom repeat library. Repeat density between male and female were estimated for races in which neo-XY sex chromosomes were present (highlighted in grey). M= male; F= female.

Species/races	P24X0	P24XY		P25XY	
Sex	M	M	F	M	F
SINE %	2.58	3.11	2.77	2.97	3.31
LINE %	24.76	26.11	26.54	28.46	28.36
LTR %	3.12	3.77	4.10	3.96	4.18
DNA transposons %	15.76	16.95	17.41	19.77	19.18
RC %	5.25	5.99	5.98	6.13	6.12
Unknown %	9.58	11.30	11.54	11.96	11.68
Satellite DNA %	7.43	4.7	2.93	2.69	2.00
Total repeat content %	68.48	71.93	71.27	75.94	74.83

Supplementary Dataset 1. Repeat consensus sequences of morabine grasshoppers. The file contains libraries generated by RepeatModeler2 (Flynn et al., 2020) and RepeatExplorer2 (Novák et al., 2020).

SI References

Flynn, J. M., Hubley, R., Goubert, C., Rosen, J., Clark, A. G., Feschotte, C., & Smit, A. F. (2020). RepeatModeler2 for automated genomic discovery of transposable element families. *Proceedings of the National Academy of Sciences*, *17*(17), 0451–9457. https://doi.org/10.1073/pnas.1921046117

Goubert, C. (2023). Assembly-Free Detection and Quantification of Transposable Elements with dnaPipeTE. In M. R. Branco & A. de Mendoza Soler (Eds.), *Transposable Elements* (Vol. 2607, pp. 25–43). Springer US. https://doi.org/10.1007/978-1-0716-2883-6_2

Goubert, C., Modolo, L., Vieira, C., ValienteMoro, C., Mavingui, P., & Boulesteix, M. (2015). De novo assembly and annotation of the Asian tiger mosquito (Aedes albopictus) repeatome with dnaPipeTE from Raw genomic reads and comparative analysis with the yellow fever mosquito (Aedes aegypti). *Genome Biology and Evolution*, *7*(4), 1192–1205. https://doi.org/10.1093/gbe/evv050

Novák, P., Neumann, P., & Macas, J. (2020). Global analysis of repetitive DNA from unassembled sequence reads using RepeatExplorer2. *Nature Protocols*, *15*(11), 3745–3776. https://doi.org/10.1038/s41596-020-0400-y

Palacios-Gimenez, O. M., Koelman, J., Palmada-Flores, M., Bradford, T. M., Jones, K. K., Cooper, S. J. B., Kawakami, T., & Suh, A. (2020). Comparative analysis of morabine grasshopper genomes reveals highly abundant transposable elements and rapidly proliferating satellite DNA repeats. *BMC Biology*, *18*(1), 199. https://doi.org/10.1186/s12915-020-00925-x

Ranallo-Benavidez, T. R., Jaron, K. S., & Schatz, M. C. (2020). GenomeScope 2.0 and smudgeplot for reference-free profiling of polyploid genomes. *Nature Communications*, *11*(1), Article 1. https://doi.org/10.1038/s41467-020-14998-3

Song, B., Marco-Sola, S., Moreto, M., Johnson, L., Buckler, E. S., & Stitzer, M. C. (2022). AnchorWave: Sensitive alignment of genomes with high sequence diversity, extensive structural polymorphism, and whole-genome duplication. *Proceedings of the National Academy of Sciences*, *119*(1), e2113075119. https://doi.org/10.1073/pnas.2113075119
